# The Romanian Translation and Cultural Adaptation of the Early Arthritis for Psoriatic Patients (EARP) Questionnaire, Psoriasis Epidemiology Screening Tool (PEST), and Toronto Psoriatic Arthritis Screen 2 (ToPAS 2)

**DOI:** 10.3390/clinpract14050168

**Published:** 2024-10-16

**Authors:** Emilia-Daniela Păsăran, Daniela Opriș-Belinski, Florian Berghea, Olguța Anca Orzan, Corina Oancea, Violeta-Claudia Bojincă, Mihai Bojincă, Denise-Ani Mardale, Ioana Cristina Saulescu, Andra-Rodica Bălănescu

**Affiliations:** 1 Faculty of Medicine, ‘Carol Davila’ University of Medicine and Pharmacy, 050474 Bucharest, Romania; daniela.opris@umfcd.ro (D.O.-B.); florian.berghea@umfcd.ro (F.B.); corina.oancea@umfcd.ro (C.O.); violeta.bojinca@umfcd.ro (V.-C.B.); mihai.bojinca@umfcd.ro (M.B.); denise-ani.mardale@drd.umfcd.ro (D.-A.M.); ioana.saulescu@umfcd.ro (I.C.S.); andra.balanescu@umfcd.ro (A.-R.B.); 2 Department of Internal Medicine and Rheumatology, ‘Sf. Maria’ Clinical Hospital, 011192 Bucharest, Romania; 3 Department of Dermatology, Elias University Emergency Hospital, 011461 Bucharest, Romania; 4 Department of Physical Medicine and Rehabilitation, ‘Carol Davila’ University of Medicine and Pharmacy, 050474 Bucharest, Romania; 5 The National Institute for Medical Assessment and Work Capacity Rehabilitation, 050659 Bucharest, Romania; 6 Department of Internal Medicine and Rheumatology, ‘Dr. Ion Cantacuzino’ Hospital, 020475 Bucharest, Romania

**Keywords:** psoriasis, psoriatic arthritis, screening tools, questionnaire, EARP, PEST, ToPAS 2, quality of life

## Abstract

Background/Objectives: Psoriasis is a chronic inflammatory condition mediated by the immune system with various manifestations. The increased prevalence of subclinical joint involvement has led to the development of early diagnostic methods for psoriatic arthritis, including several instruments that have been validated and used in clinical practice. The aim of this study was to perform the Romanian translation, cultural adaptation, and validation of three assessment tools: the Early Arthritis for Psoriatic Patients (EARP) Questionnaire, Psoriasis Epidemiology Screening Tool (PEST), and Toronto Psoriatic Arthritis Screen 2 (TOPAS 2), which are designed to evaluate early-stage arthritis in patients with psoriasis. Methods: All the activities were carried out in accordance with the internationally recognized methodology recommended by the International Society for Pharmacoeconomics and Outcome Research (ISPOR), the recommendations of the World Health Organization (WHO) regarding the translation process and the validation of instruments, and data from the international literature. These three questionnaires were administered to 29 patients with psoriasis diagnosed by biopsy. A descriptive study was conducted and the data were analyzed with appropriate statistical tests using the PSPP program. A reliability test was assessed using Cronbach’s alpha coefficient. Results: The obtained values were significant for the first two questionnaires, with a value of 0.89 for the EARP and 0.63 for the PEST, but the value was not as significant for ToPAS2, at 0.40. Conclusions: This pilot study revealed that the Romanian and original versions of the three questionnaires are similar.

## 1. Introduction

Psoriasis is a systemic disease with various manifestations that can have a significant impact on the quality of life of patients [1,2]. Over time, patients can develop psoriatic arthritis, a systemic inflammatory disorder that affects not only the skin and nails but also skeletal components such as entheses and peripheral and axial joints. In up to 70% of cases, skin lesions precede joint involvement, while in approximately 15–20% of cases, joint involvement precedes skin manifestations [3]. Approximately 40% of patients diagnosed with psoriasis will develop psoriatic arthritis, and about 15% of them will have undiagnosed psoriatic arthritis due to nonspecific joint manifestations [4]. In addition to the suffering caused by psoriasis, joint destruction and dysfunction are the final results of late diagnosis and treatment. The many negative effects in the psychological, physical, and social aspects are the main reasons why the early screening of joint involvement is necessary [5,6,7]. In this context, since no specific biomarker of the disease has been found [7,8,9,10], screening methods have been sought to assess skin and joint impairments. Over time, the focus has been placed on developing assessment tools that went through several stages of testing and improvement [6,11,12]. In 1997, the results obtained by Peloso et al. from applying the Psoriasis Assessment Questionnaire (PAQ), a 12-item questionnaire, to patients with psoriasis were published. They demonstrated a sensitivity of 85% and a specificity of 88% [13]. Five years later, Alenius et al. demonstrated 60% sensitivity and 62% specificity in a sample of 276 patients who completed the PAQ [14], prompting modifications to the questionnaire. Its sensitivity remained unchanged, while its specificity reached a value of 73% [15]. Subsequently, the members of the Group for Research and Assessment of Psoriasis and Psoriatic Arthritis (GRAPPA), based on the principle of the PAQ assessment tool [16], developed the following evaluation instruments: Psoriatic Arthritis Screening and Evaluation tool (PASE), ToPAS, and PEST [13]. Qureshi et al. created PASE, a 15-item questionnaire that assessed symptoms and articular function. Based on pilot studies, it appeared to have a specificity of 80% and a sensitivity of 93% [13,17]. In 2009, ToPAS, with 12 questions regarding the skin, nails, and joints, was evaluated by Gladman et al., and it was found to have a specificity of 93.1% and a sensitivity of 86.8% [18]. Later, Tom B.D. et al. further improved ToPAS, shaping it into ToPAS 2 and supplementing it with images representing psoriatic lesions, dactylitis, arthritis, and nail involvement. The family history of psoriasis was considered, joint conditions and morning stiffness were condensed into a single question, and the spectrum of immune-mediated rheumatic diseases was added [19]. Ibrahim et al. developed the PEST questionnaire, which consisted of five simple questions and a dummy that allowed the clinician to quickly assess the affected joints with a specificity of 78% and a sensitivity of 92% (published in 2009) [20]. Tinazzi et al. compiled another assessment tool, EARP, which they used alongside PASE as a screening method to further evaluate patients with psoriasis, proving that it has a specificity of 85% and a sensitivity of 91% (published in 2012) [21]. Due to their good sensitivity and specificity, there is a general desire to use these assessment tools in current clinical practice, and such evaluation tools have not yet been validated in Romania, though they are easy to apply and widely used [22,23,24,25]. The aim of this study is the translation, cultural adaptation, and validation in the Romanian language of these three assessment tools (EARP, PEST, and TOPAS 2). Besides the early diagnosis of joint disease and the possibility of treatment, these tools provide evidence of prevalence within a population, as well as their utility in future clinical studies in our country.

## 2. Materials and Methods

### 2.1. Design and Setting of the Study

Romanian translation, cultural adaptation, and validation were made in accordance with internationally accepted and recommended methodologies and international data from the literature regarding the process of translating and validating instruments. This study was conducted by a research team that includes rheumatologists and dermatologists (primary care physicians, specialist physicians, resident physicians), a statistician, and three translators; all team members are Romanian/English bilingual speakers. This study was approved by the Ethics Committee of Sfanta Maria Clinical Hospital in Bucharest (application number 14820/21 June 2022) and by the Ethics Committee of the Carol Davila University of Medicine and Pharmacy in Bucharest. Besides translation and cultural adaptation, in order to check the effectiveness of our work, we also conducted a pilot study over a period of six months. In the first three months, we evaluated 29 consecutive patients, representing an initial report. Additionally, we detailed the steps necessary to conduct a clinical study based on this evaluation.

### 2.2. The Questionnaires

EARP is a questionnaire with 10 questions assessing joint damage. A positive answer was evaluated with 1 point, a negative answer with 0 points, and the score was calculated by summing up the positive responses to the questions (question = Q). A score greater than 3 was considered to necessitate a rheumatological evaluation of the patient [21].

EARP = Q1 + Q2 + Q3 + Q4 + Q5 + Q6 + Q7 + Q8 + Q9 + Q10

PEST is a questionnaire with 5 questions focusing on joint involvement, with the patient able to mark painful joints on a dummy. A positive answer was evaluated with 1 point, a negative answer with 0 points, and the score is cumulative. A score equal to or greater than 3 has been considered to necessitate a rheumatologic evaluation of the patient. The score was calculated as follows [16,17,20,26]:

PEST = Q1 + Q2 + Q3 + Q4 + Q5

ToPAS 2 is a questionnaire with 13 questions focusing on skin lesions, nail involvement, and peripheral and axial joint disease. What distinguishes this questionnaire is that it provides specific images of skin, nail, and finger damage (dactylitis) to assist the patient. The score was calculated based on the affected area, as follows:

ToPAS 2 score = skin (Q1 + Q3 + Q4 + Q5) + nail (Q2) + 2 * joint (Q6 + Q7 + Q8 + Q12) + axial disease (Q9 + Q10 + Q11).

The sum corresponding to the skin domain (Q1, Q3, Q4, Q5) was evaluated with a maximum of 3 points for all positive questions. The nail area receives 1 point for a positive answer to one of the two questions (Q2a and Q2b). The sum corresponding to the joint area (Q6, Q7, Q8, Q12) was evaluated with a maximum of 3 for all positive questions. In the case of the spine domain (Q9, Q10, Q11), the maximum score is 2 if several questions have a positive answer and 0 if none of them have a positive answer. A score of 8 or more was considered to require a rheumatologic evaluation of the patient. In any case, if the joint area score is 2, it was considered to potentially warrant a rheumatologic evaluation [18,19]. Data from the literature indicate the high sensitivity and specificity of the questionnaires when used together [27].

### 2.3. Description of All Processes, Interventions and Comparisons

**Figure 1:**
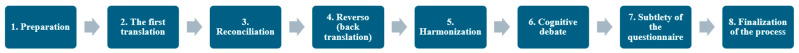
The method.

The study protocol needs to be simple and concise, addressing an audience of varying educational backgrounds and different ages. The recommended stages to be followed, according to the translation guidelines, are described in Figure 1 [28,29,30].

In order to apply the described methods, the first step taken was obtaining permission from the authors both for the translation and protocol development. Then, the research team was formed, approval was obtained from the ethics committees of the hospitals where the study was carried out, the translators were chosen, and the concept of the study was explained. The second step was to complete the initial translations from English to Romanian. The committee convened, and linguistic nuances were discussed (the third step). The fourth step, the reverse stage, involved a native English speaker translating the materials from Romanian into English. The research team convened, and differences were discussed. In the fifth step, the Romanian version and the English version obtained were analyzed and compared with the originals, with no major differences observed. After each translation, the research team met, and discrepancies were discussed. Differences were analyzed and adapted for the Romanian versions. In the sixth step, the cognitive debriefing stage, the team trained specifically for this task and administered the questionnaires to a group of 29 psoriasis patients in the dermatology clinic. The results obtained were recorded in a written report. In the seventh step, issues were discussed, and optimal solutions were sought. Also, necessary modifications were made, and a new Romanian version was obtained. The latest Romanian version of the questionnaires was tested on another similar batch of patients and can be found in Appendix A.

### 2.4. Selection of Patients

The pilot study was carried out in the Department of Dermatology of the Elias Emergency University Hospital in Bucharest with the help of dermatologists, including primary care doctors and resident doctors. The patients included in the study were diagnosed with psoriasis by dermatologists through skin biopsy. They were aged between 18 and 80, from all socio-economic backgrounds, and native Romanian speakers. Exclusion criteria considered patients with cognitive deficits and patients who were unable to write or read. The informed consent form for study participation and the consent form for personal data processing were explained to the patients and signed by them. The questionnaires were completed by 29 consecutive patients over a period of three months in the presence of medical staff. Patients were asked if they understood the questions, if there were any words or phrases they did not understand or found offensive or unacceptable, if there were alternatives they preferred, or if certain words or phrases inspired them. The results obtained were discussed by the team members. Optimal solutions were found to refine the questionnaires and obtain the modified versions, which were tested again, using the same method, on another group of patients. It was observed that the changes made were much more accessible to them. After completing this process, the team decided on the final versions of EARP, PEST, and ToPAS 2.

### 2.5. Statistical Analysis

A descriptive study was performed and the data were processed with appropriate statistical tests using the PSPP version 3, 2007. To obtain a general overview of demographics and the distribution of responses to questions, percentages, and frequencies were evaluated. Numerical variables (such as age) were analyzed using Independent Sample T-tests, while categorical variables (such as gender, place of origin, and educational level) were analyzed using chi-squared tests. A 95% confidence interval was applied to the variables, and statistical significance was reported at *p* < 0.05. The reliability test was assessed using Cronbach’s alpha coefficient.

## 3. Results

The mean age of the study group was 51 (42–59.50) years. The male-to-female ratio was 17/12, where the average age for the women was 52.50 (42.50–60.25) years and 44 (39–64) years for the men. Our patients were mainly from urban areas and most had a medium level of education (Table 1).

### 3.1. The Questionnaire Analysis

For the questions where the level of understanding was below 100%, the level of understanding was analyzed based on gender, place of origin, and level of education.

#### 3.1.1. EARP

In the case of the EARP questionnaire, the first and sixth questions presented difficulties in terms of understanding. For Q1, “Do your joints hurt?**/**Va dor articulatiile?”, regarding the understanding of joint pain, the patients associated the pain with mechanical causes, such as injury or trauma. In the case of Q6, “Do your wrists and fingers swell?**/**Vi se umfla incheieturile pumnilor sau degetele mainii?”, patients associated joint swelling with subcutaneous cellular edema. In the statistical analysis of the EARP questionnaire, a good level of understanding was observed; most of the questions were understood by all the subjects, except for Q1 and Q6. However, the statistical analysis showed that the meaning of the questionnaires was not influenced by gender or place of origin, but it seemed to be influenced by the level of education, considering that this parameter approached the threshold of statistical significance (*p* = 0.07).

#### 3.1.2. PEST

In the first part of the PEST test, aimed at locating painful joints on a dummy, 25 patients (86.21%) had no difficulty completing the dummy task, while 4 patients (13.79%) encountered difficulty. In the statistical analysis of PEST, a good level of understanding was observed, but only Q1, “Have you ever had a swollen joint (or joints)? “**/**”Ati avut vreodata una sau mai multe articulatii umflate?”, and Q3, “Do your finger nails or toe nails have holes or pits? “**/**”Unghiile de la maini sau picioare prezinta aspect de unghie intepata cu acul?”, presented difficulty. However, the level of understanding was not influenced by gender, place of origin, or level of education.

#### 3.1.3. ToPAS 2

In the case of the ToPAS 2 questionnaire, difficulties were encountered in understanding the following questions. Q2: “Have you ever noticed any of these changes in your fingernails: (a) Pits in the nails as shown in this picture? If yes, have you ever seen a doctor about this? What was the diagnosis? (b) Lifting of the nail from the nail bed as shown in this picture? If yes, have you ever seen a doctor about this? or I don’t know. “**/**”Ati observant vreodata oricare din urmatoarele modificari la nivelul unghiilor astfel: (a) Aspect de unghie intepata cu acul? Daca DA: Ati consultat un medic? Care a fost diagnosticul? Sau Nu stiu. (b) Ati observant vreodata departarea unghiei de patul unghial? Daca DA: Ati consultat un medic?Care a fost diagnosticul? Sau Nu stiu”; Q9: “Have you ever had neck pain and stiffness lasting at least 3 months that was not the result of injury? If yes, have you ever seen a doctor about this? What was the diagnosis? or I don’t know. ”**/**”Ati avut vreodata durere la nivelul gatului si intepeneala care sa dureze cel putin 3 luni si care sa nu fie rezultatul unui traumatism? Daca DA: Ati consultat un medic? Care a fost diagnosticul? Sau Nu stiu”; Q11: “Have you ever had neck or back pain that got better with activity but was worsened by rest? If yes, have you ever seen a doctor about this? What was the diagnosis? or I don’t know. “**/**”Ati avut vreodata durere de gat sau de spate (de sale) care s-a imbunatatit cu miscarea dar s-a inrautatit in repaus? Daca DA: Ati consultat un medic? Care a fost diagnosticul? Sau Nu stiu”; and Q13: “Have you ever been diagnosed with any of the following conditions? If yes, which ones? Psoriatic arthritis, Ankylosing Spondylitis, Reiter’s syndrome, Uveitis, Inflammatory bowel disease, Rheumatic arthritis, Juvenile idiopathic arthritis, Osteoarthritis, Fibromyalgia, Lupus (SLE), Scleroderma, Connective tissue disease, other. “**/**”Ati fost vreodata diagnosticat cu vreuna din urmatoarele boli?Artrita psoriazica, Spondilita anchilozanta, Sindrom Reiter, Uveita, Boala inflamatorie intestinala, Poliartrita reumatoida, Artrita idiopatica juvenila, Artroza, Fibromialgie, Lupus eritematos sistemic, Sclerodermie, Boala de tesut conjunctiv, Altele (Specificati)”. The understanding based on gender did not impact questions 2, 9, 11, or 13. However, concerning the place of origin, Q13 was understood by one-third of those from rural areas (33.33%) and by all 26 subjects from urban areas (100%), with a significant statistical value of *p* = 0.007. The reliability was tested with Cronbach’s alpha coefficient, which assesses the degree of correlation between the items and the ability of the items to assess the studied phenomenon. The obtained values were significant for the first two questionnaires, at 0.89 for EARP and 0.63 for PEST, but the values were weaker for ToPAS2, at 0.40.

## 4. Discussion

This is a preliminary study as part of the process of validating three instruments in Romania for the assessment of early arthritis in patients with psoriasis. The obtained results showed variability in the outcomes, yet with good reliability. At this stage, we have not found major difficulties in either following the steps outlined in the international recommendations or the doctor–patient relationship. This study focused on the patient’s understanding of the translated terms and phrases. It was divided into two practical phases: the first phase involved the administration of the questionnaires to a group of 29 patients, and the second phase involved the reassessment, modification, and subsequent distribution of the questionnaires, again to a group of 29 patients. The first issue flagged was the method of completing the questionnaire responses, which is why in each questionnaire, marking the boxes with an “X” is specified. In the case of the EARP questionnaire, for question number 3, to assist the patient, the more familiar phrase “the small of the back” was added to “low back pain”, and it was also used in the final form of the tool.

**Figure 2:**
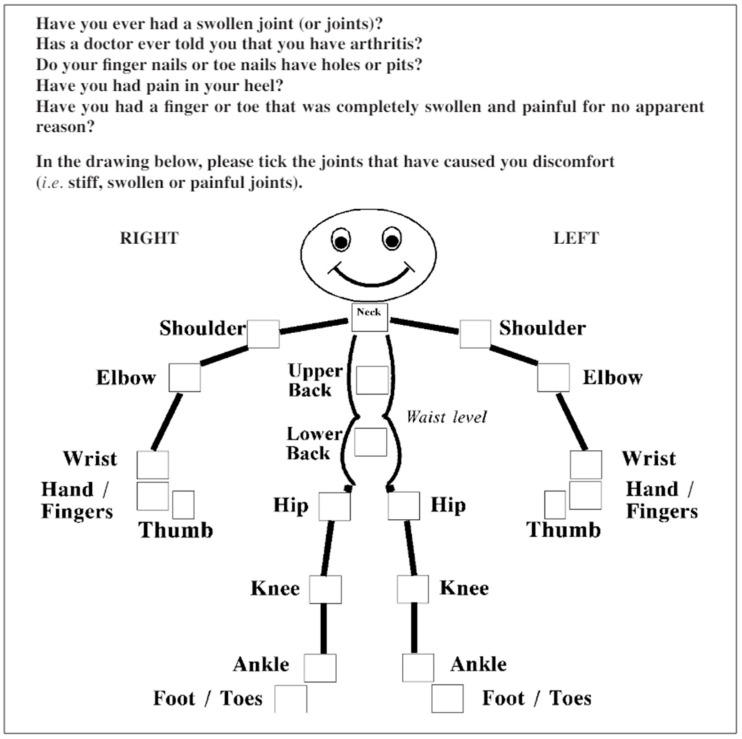
The PEST questionnaire [25].

**Figure 3:**
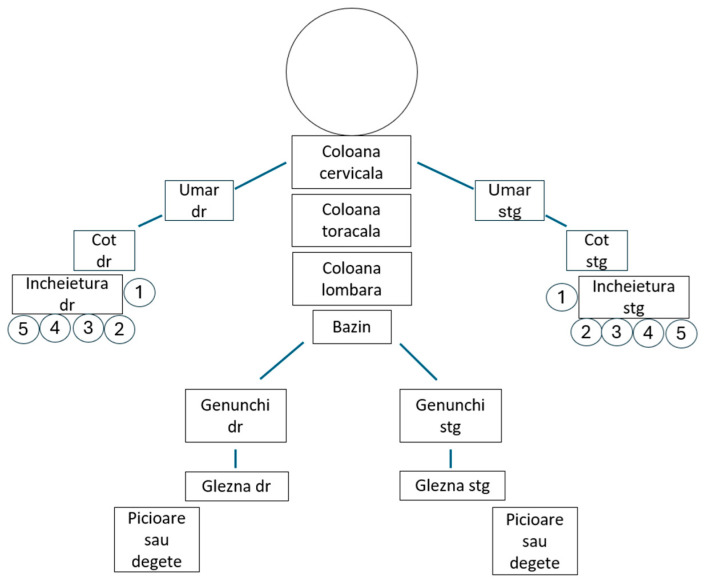
The dummy.

The PEST questionnaire also posed cultural adaptation difficulties in question number 3, but these were easily resolved. Additionally, the use of the dummy posed issues for the patients in identifying painful joints; thus, a simpler version was chosen, which aligned the joints within the represented segment for easier marking (Figure 2 and Figure 3).

In the case of the ToPAS 2 questionnaire, difficulty was observed in tracking the structure of the questions. Therefore, a more accessible arrangement/order was opted for. During the adaptation stage, in questions 9 and 11, “la nivelul gatului” (neck pain) was mistaken for odynophagia, so the more familiar expressions “la nivelul cefei” and “de ceafa”, which mean “neck pain”, were added. Also, during the adaptation stage, “de șale” (the small of the back) was added to question number 10, which was retained in the final form of the questionnaire. Relating to the last question, number 13, details of the osteoarthritis diagnosis such as the presence of spondylosis, coxarthrosis, or gonarthrosis, were added, as the patient was not familiar with the spectrum of conditions included in the term osteoarthritis.

Regarding the statistical analysis of these three questionnaires, no influence of gender was observed on the patients’ understanding and response to the questions. However, the patient’s background and level of education might have a role in the simplicity or complexity of understanding certain words, hence the need for clarification. Good internal consistency was found for the EARP and PEST questionnaires but this was weaker for ToPAS 2. This may have several explanations. We expect that the reliability will increase with a larger patient cohort. ToPAS 2 had the highest percentage of questions that were difficult to understand. In addition, the order of questionnaire completion and fatigue when responding to questions after completing the first two could have influenced the results. These findings allow us to improve our methodology in the validation stage of the questionnaires.

This study also has some limitations that may be encountered in the future, such as systematic errors like the subjectivity of the questionnaires; there may be underestimation or overestimation of joint symptomatology. One important limitation was the small number of participants in this pilot study, which is necessary to lay the foundation for future large-scale studies. Therefore, an evaluation of the procedure and the methods used along with the error-detection technique and further necessary adjustments prove a must. The time allowed for the first practical phase was limited. Consequently, the validation phase will require a longer period of time as well as a larger number of patients in order to demonstrate the psychometric properties of the questionnaires.

The need to validate and implement these three questionnaires in current clinical practice in our country originates from the patients’ needs for early treatment. Since psoriasis precedes the joint disease, identifying patients with psoriatic arthritis proves an important factor. Delaying the destructive processes and good communication between clinicians are also important. The lack of biomarkers has made these evaluation tools useful for future research in this field. However, the need to be culturally adapted and validated in many countries is important in order to benefit from international evaluation and provide a more thorough understanding of the topic.

## 5. Conclusions

This study reveals that the Romanian and original versions of the three questionnaires are similar. There were some minor difficulties in the study’s execution, but they were manageable, resulting in simpler versions of the questionnaires in terms of question arrangement and understanding. Therefore, the Romanian versions of the three questionnaires for assessing joint disease in patients with psoriasis are suitable for clinical practice and research purposes. The decision to start with a pilot study was made to test the feasibility and the methods needed to support a study with a larger number of participants. The results provide valuable guidance for further validation of the questionnaire under discussion, which will broaden our perspective on the relationship between pre-clinical and clinical aspects of psoriatic arthritis, on the one hand, and the assessment tools such as questionnaires, on the other.

## Figures and Tables

**Table 1:** Sociodemographic variables.

Variables	N	%
Sex - Men - Women	17 12	59 41
Origin environment - Urban area - Rural area	26 3	90 10
Studies - Basic - Medium - High	2 21 6	7 72 21
Age, years—median (IQR *) 51 (42–59.50) Men 44 (39–64) Women 52.50 (42.50–60.25)	Age (mean ± SD, years) 49.79 (±13.82) Men 49.53 (±13.56) Women 50. 17 (±11.56)	

* IQR—interquartile range.

## Data Availability

Please contact the corresponding author for any inquiries regarding data access.

## Appendix A

Early Arthritis for Psoriatic Patients Questionnaire (EARP)

Va rugam sa raspundeti la urmatoarele intrebari cu DA sau NU prin bifarea casutelor cu X.

	**DA**	**NU**
1. Va dor articulatiile?		
2. Ati luat medicamente antiinflamatoare pentru dureri articulare mai mult de 2 ori pe saptamana in ultimele 3 luni?		
3. Va treziti noaptea din somn din cauza durerii de spate joasa (de sale)?		
4. Simtiti intepeneala la nivelul mainilor, dimineata la trezire, pentru mai mult de 30 de minute?		
5. Va dor incheieturile pumnilor sau degetele mainii?		
6. Vi se umfla incheieturile pumnilor sau degetele mainii?		
7. V-a durut si vi s-a umflat vreun deget mai mult de 3 zile?		
8. Vi se umfla tendonul lui Achile?		
9. Va dor labele picioarelor sau gleznele?		
10. Va dor coatele sau soldurile?		
Da = 1 punct, Nu = 0 puncte, Total = ( ) din 10 puncte.		

Psoriasis Epidemiology Screening Tool (PEST)

In desenul de mai jos, va rugam, bifati cu X articulatiile care v-au cauzat disconfort (articulatii dureroase, umflate, rigide sau intepenite).

	**DA**	**NU**
1. Ati avut vreodata una sau mai multe articulatii umflate?		
2. V-a spus vreun medic, vreodata, ca aveti artrita?		
3. Unghiile de la maini sau picioare prezinta aspect de unghie intepata cu acul?		
4. Ati avut vreodata durere in calcai?		
5. Ati avut vreun deget de la maini sau picioare complet umflat sau dureros fara vreun motiv anume?		

Va rugam sa raspundeti la urmatoarele intrebari cu Da sau Nu prin bifarea cu X a casutelor aferente.

Fiecare intrebare al carui raspuns este DA valoreaza 1 punct. Se calculeaza suma intrebarilor. Total = ( ) din 5 puncte.

Toronto Psoriatic Arthritis Screen 2 (ToPAS 2)

Va rugam sa bifati cu X, DA, NU sau Nu stiu la urmatoarele intrebari:

1. Ati avut vreodata pe piele o eruptie constand in zone rosiatice si cu scuame albe-argintii asemanatoare celor din poza (eruptia putand fi localizata pe orice zona a corpului)? Department of Dermatology, Elias University Emergency Hospital, Bucharest, Romania	DA Daca DA: Ati consultat un medic legat de aceasta? Care a fost diagnosticul?	NU	Nu stiu
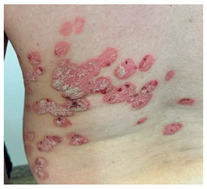
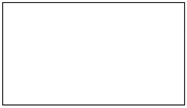
2. Ati observant vreodata oricare din urmatoarele modificari la nivelul unghiilor astfel: (a) Aspect de unghie intepata cu acul? Hochberg MC, Gravallese EM, Silman AJ, et al. Clinical features of psoriatic arthritis. Rheumatology, vol. 1. 7th ed., Philadelphia: Elsevier; 2019, p. 1054. [31]	DA Daca DA: Ati consultat un medic? Care a fost diagnosticul?	NU	Nu stiu
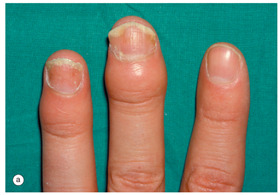
(b) Ati observant vreodata ridicarea unghiei de pe patul unghial? Hochberg MC, Gravallese EM, Silman AJ, et al. Clinical features of psoriatic arthritis. Rheumatology, vol. 1. 7th ed., Philadelphia: Elsevier; 2019, p. 1053. [31]	DA Daca DA: Ati consultat un medic? Care a fost diagnosticul?	NU	Nu stiu
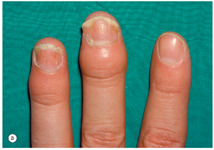
3. Ati consultat vreodata un medic pentru eruptie pe piele?	DA Daca DA: Ati consultat un medic pentru aceasta eruptie descrisa mai sus? Care a fost diagnosticul?	NU	Nu stiu
4. Ati fost diagnosticat cu Psoriazis de catre un medic vreodata ?	DA	NU	
5. A avut cineva vreodata, in familia dumneavoastra, Psoriazis?	DA	NU	
6. Ati avut vreodata durere sau intepeneala in articulatii (la degetele mainilor sau picioarelor, incheietura mainii, glezne) care sa nu fie rezultatul unui traumatism?	DA Daca DA: Ati consultat un medic? Care a fost diagnosticul?	NU	Nu stiu
7. Ati avut vreodata articulatii umflate si rosii, la fel ca in imagine, care sa nu fie rezultatul unui traumatism? Hochberg MC, Gravallese EM, Silman AJ, et al. Clinical features of juvenile idiopathic arthritis. Rheumatology, vol. 1. 7th ed., Philadelphia: Elsevier; 2019, p. 884. [32]	DA Daca DA: Ati consultat vreun medic? Care a fost diagnosticul?	NU	Nu stiu
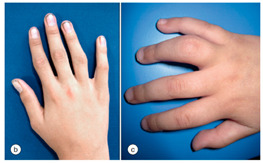
8. Ati avut vreodata vreun deget de la mana sau picior rosu si umflat in forma de “carnacior” la fel ca in imagine, care sa nu fie rezultatul unui traumatism? Hochberg MC, Gravallese EM, Silman AJ, et al. Clinical features of psoriatic arthritis. Rheumatology, vol. 1. 7th ed., Philadelphia: Elsevier; 2019, p. 1053. [31]	DA Daca DA: Ati consultat un medic? Care a fost diagnosticul?	NU	Nu stiu
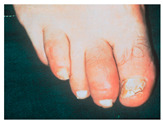
9. Ati avut vreodata durere si intepeneala la nivelul cefei care sa dureze cel putin 3 luni si care sa nu fie rezultatul unui traumatism?	DA Daca DA: Ati consultat vreun medic? Care a fost diagnosticul?	Nu	Nu stiu
10. Ati avut vreodata durere de spate (de sale) si intepeneala cu o durata de cel putin 3 luni, care sa nu fie rezultatul unui traumatism?	DA Daca DA: Ati consultat vreun medic? Care a fost diagnosticul?	NU	Nu stiu
11. Ati avut vreodata durere de ceafa sau de spate (sale) care s-a imbunatatit cu miscarea dar s-a inrautatit in repaus?	DA Daca DA: Ati consultat un medic? Care a fost diagnosticul?	NU	Nu stiu
12. Ati consultat vreodata un medic pentru durere articulara?	DA	NU	
13. Ati fost vreodata diagnosticat cu vreuna din urmatoarele diagnostice? Bifati casutele! □ Artrita psoriazica □ Spondilita anchilozanta □ Sindrom Reiter □ Uveita □ Boala inflamatorie intestinala □ Artrita reumatoida □ Artrita idiopatica juvenila □ Artroza (Spondiloza, Coxartroza, Gonartroza) □ Fibromialgie □ Lupus eritematos systemic □ Sclerodermie □ Boala de tesut conjunctiv □ Altele(Specificati)	DA	NU	Nu stiu
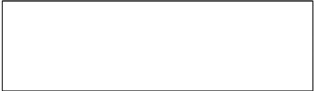

ToPAS 2 score = skin (Q1 + Q3 + Q4 + Q5) + nail (Q2) + 2 * joint (Q6 + Q7 + Q8 + Q12) + axial disease (Q9 + Q10 + Q11).
